# Hyperbaric Oxygen Reduces Oxidative Stress Impairment and DNA Damage and Simultaneously Increases HIF-1α in Ischemia–Reperfusion Acute Kidney Injury

**DOI:** 10.3390/ijms25073870

**Published:** 2024-03-30

**Authors:** Jelena Nesovic Ostojic, Sanjin Kovacevic, Milan Ivanov, Predrag Brkic, Maja Zivotic, Nevena Mihailovic-Stanojevic, Danijela Karanovic, Una Jovana Vajic, Rada Jeremic, Djurdjica Jovovic, Zoran Miloradovic

**Affiliations:** 1 Department of Pathological Physiology, Faculty of Medicine, University of Belgrade, 11000 Belgrade, Serbia; sanjin.kovacevic@med.bg.ac.rs; 2 Department of Cardiovascular Physiology, Institute for Medical Research, National Institute of Republic of Serbia, University of Belgrade, 11000 Belgrade, Serbia; ivmilan@imi.bg.ac.rs (M.I.); nevena@imi.bg.ac.rs (N.M.-S.); danijela.karanovic@imi.bg.ac.rs (D.K.); unajovana@imi.bg.ac.rs (U.J.V.); djurdjica@imi.bg.ac.rs (D.J.); zokim@imi.bg.ac.rs (Z.M.); 3 Department of Medical Physiology, Faculty of Medicine, University of Belgrade, 11000 Belgrade, Serbia; predrag.brkic@med.bg.ac.rs (P.B.); rada.jeremic@med.bg.ac.rs (R.J.); 4 Institute of Pathology, Faculty of Medicine, University of Belgrade, 11000 Belgrade, Serbia; maja.zivotic@med.bg.ac.rs

**Keywords:** oxidative stress, FRAP, 3-nitrotyrosine, γH2AX(S139), HIF-1α, NF-κB, spontaneously hypertensive rats, HBO preconditioning

## Abstract

The central exacerbating factor in the pathophysiology of ischemic–reperfusion acute kidney injury (AKI) is oxidative stress. Lipid peroxidation and DNA damage in ischemia are accompanied by the formation of 3-nitrotyrosine, a biomarker for oxidative damage. DNA double-strand breaks (DSBs) may also be a result of postischemic AKI. γH2AX(S139) histone has been identified as a potentially useful biomarker of DNA DSBs. On the other hand, hypoxia-inducible factor (HIF) is the “master switch” for hypoxic adaptation in cells and tissues. The aim of this research was to evaluate the influence of hyperbaric oxygen (HBO) preconditioning on antioxidant capacity estimated by FRAP (ferric reducing antioxidant power) and ABTS (2,2′-azino-bis(3-ethylbenzothiazoline-6-sulfonic acid)) assay, as well as on oxidative stress parameter 3-nitrotyrosine, and to assess its effects on γH2AX(S139), HIF-1α, and nuclear factor-κB (NF-κB) expression, in an experimental model of postischemic AKI induced in spontaneously hypertensive rats. The animals were divided randomly into three experimental groups: sham-operated rats (SHAM, *n* = 6), rats with induced postischemic AKI (AKI, *n* = 6), and group exposed to HBO preconditioning before AKI induction (AKI + HBO, *n* = 6). A significant improvement in the estimated glomerular filtration rate, eGFR, in AKI + HBO group (*p* < 0.05 vs. AKI group) was accompanied with a significant increase in plasma antioxidant capacity estimated by FRAP (*p* < 0.05 vs. SHAM group) and a reduced immunohistochemical expression of 3-nitrotyrosine and γH2AX(S139). Also, HBO pretreatment significantly increased HIF-1α expression (*p* < 0.001 vs. AKI group), estimated by Western blot and immunohistochemical analysis in kidney tissue, and decreased immunohistochemical NF-κB renal expression (*p* < 0.01). Taking all of these results together, we may conclude that HBO preconditioning has beneficial effects on acute kidney injury induced in spontaneously hypertensive rats.

## 1. Introduction

Acute kidney injury (AKI) is defined as a sudden decrease in glomerular filtration rate (GFR), which includes structural damage and loss of function [1], leading to azotemia and often oliguria or anuria. In hospital settings, AKI commonly occurs in patients who underwent cardiac and other complex surgery [2], sepsis [3], renal transplantation [4], and reparation of an aneurysm [5]. The main risk factors include diabetes, hypertension, and peripheral artery disease [6]. AKI is also recognized as an increasing healthcare challenge [3], due to its rising incidence, complex pathophysiology, and limited treatment. A large number of studies have been directed at elucidating pathogenesis and developing AKI therapeutics in animal models [7,8]. However, to date, none of these therapies have been translated into clinical practice [9].

The most common cause of AKI is renal ischemia–reperfusion injury (IRI) [10]. Several mechanisms are involved in the pathophysiology of IRI, but the literature data confirm that oxidative stress plays a crucial role in this process and is recognized as a central deleterious factor [11,12] especially in the reperfusion phase, when the most IRI damage might occur.

An increased level of reactive oxygen species (ROS) that cannot be regulated by endogenous antioxidants promotes inflammation, vascular dysfunction, and renal tubule cell cytotoxicity, all of which are observed in the pathogenesis of AKI [13]. In addition, DNA damage, including physical DNA double-strand breaks (DSBs), and oxidative base modifications may also be a result of renal IRI [14]. Gama H2AX is the phosphorylated form of the histone H2AX at serine 139 (γH2AX(S139)), [15] and because of its sensitivity and utility for the detection of DNA double-strand breaks, γH2AX(S139) has been identified as a potentially useful biomarker with clinical implications [16]. Previously, in our investigation, we showed that γH2AX(S139) may be used to indicate DNA damage that follows ischemia–reperfusion AKI [17]. On the other hand, lipid peroxidation and DNA damage in ischemia are associated with the formation of 3-nitrotyrosine, a biomarker for ROS/reactive nitrogen species (RNS), suggesting that NO^•^, O_2_^•−^, and/or peroxynitrite, contribute to renal oxidative damage [18,19]. In addition, postischemic AKI is accompanied by inflammation [20], and nuclear factor κB (NF-κB) serves as a central mediator of inflammatory response [21].

At the same time, cellular hypoxia is one of the most powerful inducers of gene expression, metabolic changes, and regenerative processes [22] in order to survive unfavorable hypoxic conditions. Hypoxia-inducible factor (HIF) is the “master switch” for hypoxic adaptation in cells and tissues. In kidneys, as in other organs, HIF improves the tolerance to conditions of hypoxia or ischemia in vivo, but HIF is often suboptimal under these conditions. Thus, there is a great therapeutic potential to activate or target HIF [23].

The therapeutical basis of hyperbaric oxygenation (HBO) is to create a hyperbaric environment with pure oxygen that allows for a significant increase in the oxygen supply to blood and to the tissues even without the Hb involvement [24], so it could be used to fix tissue hypoxia and chronic hypoxemia and to help in the clinical approach to reperfusion injuries [25]. In contrast to hypoxia, the human body has not developed specific adaptative mechanisms to hyperoxia. Moreover, the exposure to intermittent hyperoxia activates cellular mechanisms and mediators which are induced by hypoxia [22]. Actually, similar with intermittent hypoxia, the exposure to short-term hyperoxia may provoke favorable outcomes in the cell. This led us to the idea that hyperbaric oxygenation preconditioning may be beneficial in cases with a high risk of developing postischemic AKI. High PO_2_ in the tissues during HBO also leads to an increased production of ROS and RNS that may have important implications in the cellular signaling, resulting in the synthesis of different growth factors, thus improving neovascularization and showing immunomodulatory properties [26,27], which contribute to clinical efficacy of hyperbaric oxygenation. Moreover, HBO therapy upregulates HIF via ROS/RNS and Extracellular Regulated Kinases (ERK1/ERK2) pathways [22,28].

In our previous studies performed on the same experimental model of postischemic AKI, we showed that hyperbaric oxygen used for preconditioning may be beneficial on the pathogenesis of acute kidney injury [29,30,31], and now we wanted to go further into elucidating these protective effects with the aim of this research to evaluate the influence of hyperbaric oxygen (HBO) preconditioning on antioxidant capacity estimated via a FRAP (ferric reducing antioxidant power) and ABTS (2,2′-azino-bis(3-ethylbenzothiazoline-6-sulfonic acid) assay, as well as on oxidative stress parameter 3-nitrotyrosine, and to gain more insights into the mechanisms of HBO preconditioning by assessing its effects on γH2AX histone, HIF-1α, and NF-κB expression in an experimental model of postischemic AKI induced in spontaneously hypertensive rats.

## 2. Results

### 2.1. Estimated Glomerular Filtration Rate (eGFR)

**Figure 1:**
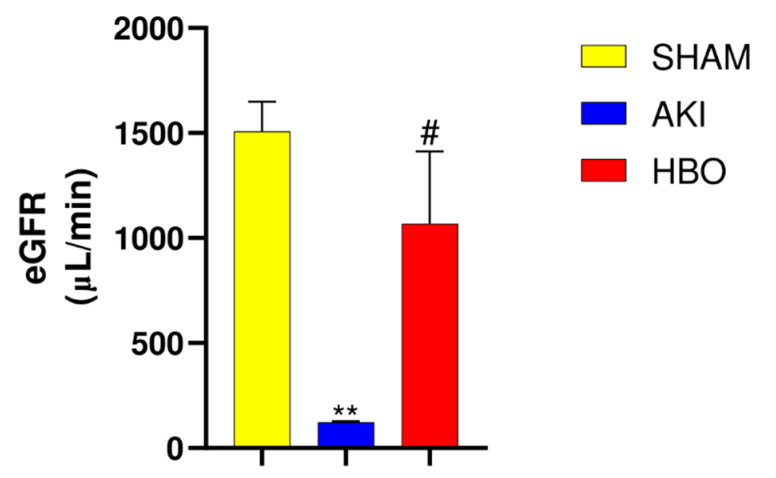
Estimated glomerular filtration rate in different experimental groups. One-way ANOVA with Tukey’s post hoc test: ** *p* < 0.01 vs. SHAM group; # *p* < 0.05 vs. AKI group.

In order to evaluate the experimental procedure, the eGFR was calculated. In the group with induced postischemic AKI, a significantly decreased eGFR was noticed in comparison to the sham-operated rats (*p* < 0.001, Figure 1), while in group with HBO preconditioning, eGFR was significantly increased in comparison to the AKI group (*p* < 0.05, Figure 1).

### 2.2. Antioxidant Capacity of Plasma

**Figure 2:**
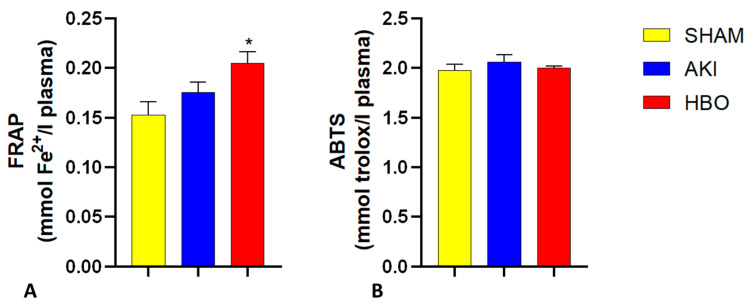
Antioxidant capacity of plasma, estimated via FRAP (**A**) and ABTS (**B**) assay in different experimental groups. One-way ANOVA with Tukey’s post hoc test: * *p* < 0.05 vs. SHAM group.

Regarding the antioxidant capacity of plasma, estimated with a FRAP assay (Figure 2A) and ABTS assay (Figure 2B), no significant difference was found among the groups in this experimental setting, with the exception that the group with HBO preconditioning had a significantly increased antioxidant capacity, when considering the FRAP assay in comparison to SHAM-operated rats.

### 2.3. Immunohistochemical Analysis

#### 2.3.1. 3-Nitrotyrosine (3-NT) Expression

**Figure 3:**
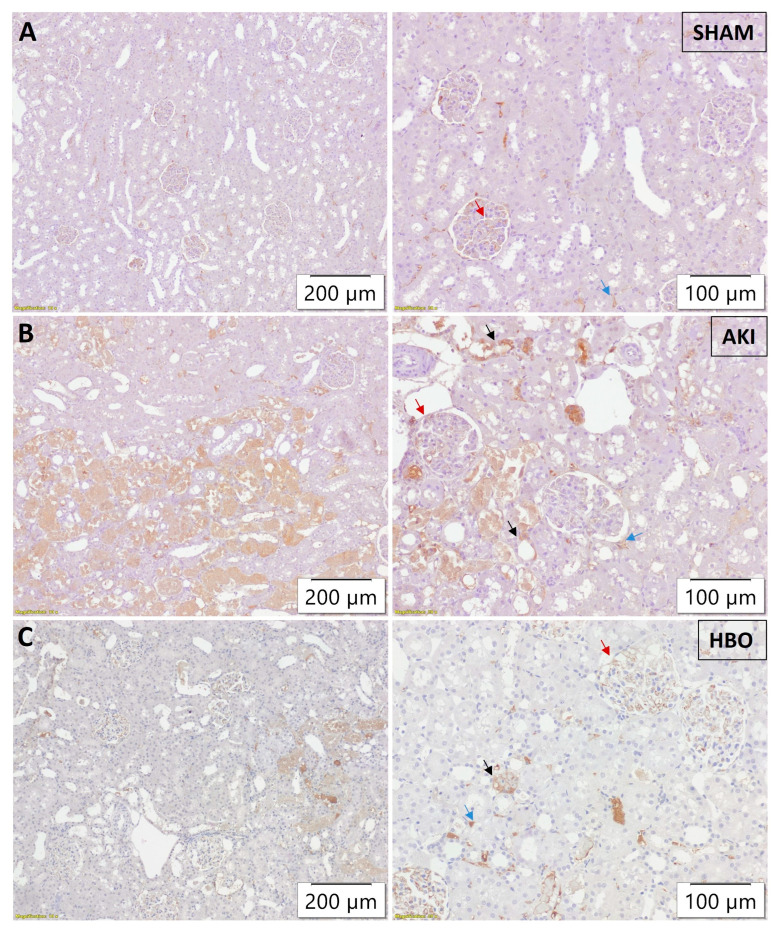
Immunohistochemical 3-NT expression in glomeruli (red arrows), tubules (black arrows), and peritubular capillaries (blue arrows) in representative kidney samples collected in different experimental groups: SHAM (**A**), AKI (**B**), and HBO (**C**).

In SHAM-operated rats (Figure 3A), 3-NT expression was minimal, only focally present in peritubular capillaries and glomeruli, without tubular expression. In the AKI group, strong and abundant tubular expression of 3-NT, along with expression in glomeruli and peritubular capillaries, was noticed (Figure 3B). HBO preconditioning led to an evident decrease in 3-NT intensity and the extent of expression in all of the aforementioned structures (Figure 3C).

#### 2.3.2. Histone γH2AX(S139) Expression

**Figure 4:**
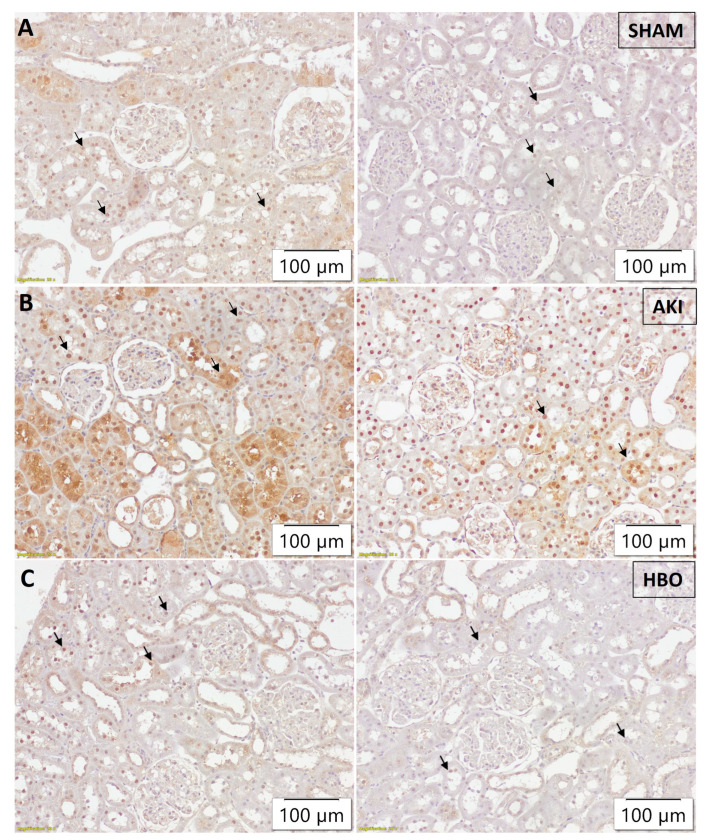
Immunohistochemical histone γH2AX(S139) expression in the nuclei of proximal tubular epithelial cells (arrows) in subcortical and middle cortical area of representative kidney samples collected in different experimental groups: SHAM (**A**), AKI (**B**), and HBO (**C**).

In the SHAM-operated group, histone γH2AX(S139) was expressed in the nuclei of proximal tubular epithelial cells in cortical kidney parenchyma to varying extents, mostly pronounced in subcapsular cortical area, with decreased expression in the middle cortical zone, and even more decreased in the juxtamedullary cortical zone. Since the medulla does not contain proximal tubules, this kidney zone did not express γH2AX(S139) (Figure 4A). In the AKI group, an evidently higher extent of expression was detected in the nuclei of cortical proximal tubular cells, with more pronounced expression in the subcapsular region, middle cortex, and juxtamedullary cortical zone (Figure 4B) in comparison to the control group. HBO preconditioning led to an evident decrease in γH2AX(S139) expression in all observed cortical structures (Figure 4C), with expression more pronounced in comparison to the SHAM-operated rats, but less abundant and present in less proximal tubular epithelial cells in comparison to the AKI group.

#### 2.3.3. NF-κB Expression

**Figure 5:**
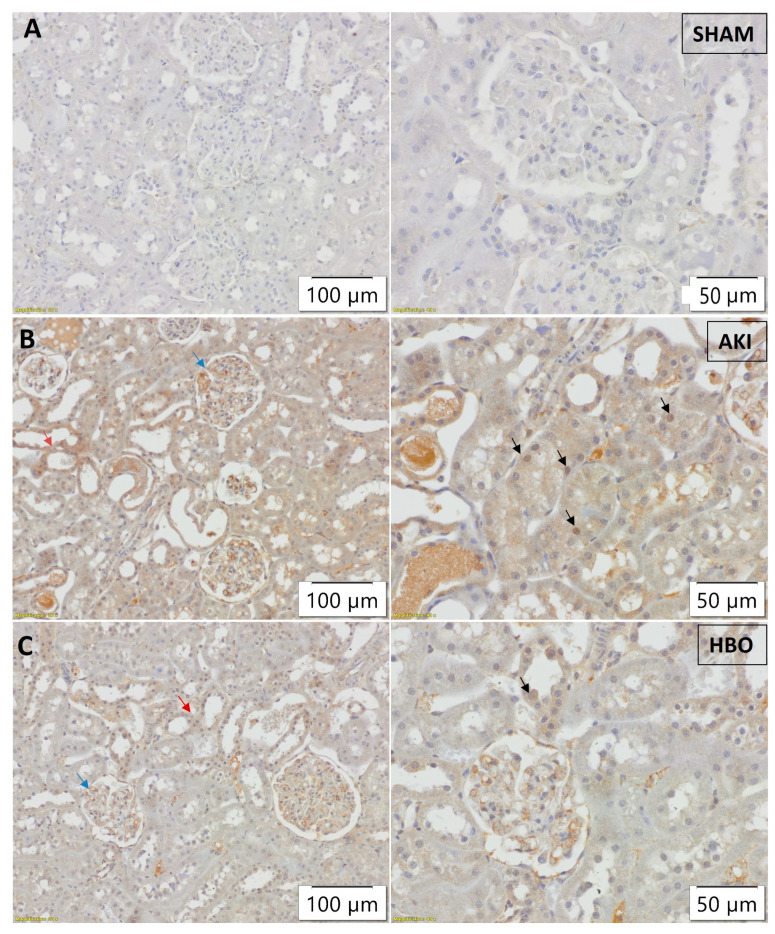
Immunohistochemical NF-κB expression in glomeruli (blue arrows), tubules (red arrows), and nuclei of tubular epithelial cells (black arrows) in representative kidney samples collected in different experimental groups: SHAM (**A**), AKI (**B**), and HBO (**C**).

In SHAM-operated rats (Figure 5A), NF-κB expression was minimal, only focally present in peritubular capillaries and glomeruli, without tubular expression. In the AKI group, strong and abundant nuclear and cytoplasmatic tubular expression, along with expression in glomeruli and peritubular capillaries, was noticed (Figure 5B). In the group with HBO preconditioning evident, decreases in the intensity and extent of expression in all of the aforementioned structures were noticed (Figure 5C).

**Figure 6:**
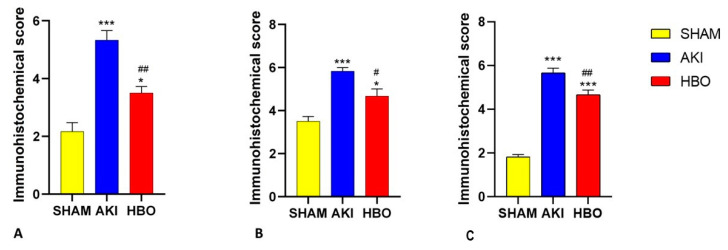
Immunohistochemical score of 3-nitrotyrosine (3-NT) (**A**), histone γH2AX(S139) (**B**), and NF-κB (**C**) expression in different experimental groups. One-way ANOVA with Tukey’s post hoc test: *** *p* < 0.001; * *p* < 0.05 vs. SHAM group; ## *p* < 0.01; # *p* < 0.05 vs. AKI group.

In order to evaluate the obtained results, a semi-quantitative analysis was performed. The immunohistochemical score of 3-NT (Figure 6A), histone γH2AX(S139) (Figure 6B), and NF-κB (Figure 6C) expression was significantly higher in the AKI group compared to control group (3-NT, *p* < 0.001; γH2AX(S139), *p* < 0.001; NF-κB, *p* < 0.001). In comparison with the AKI group, the immunohistochemical scores for both parameters were significantly lower for the group with hyperbaric oxygen preconditioning (3-NT, *p* < 0.01; γH2AX(S139), *p* < 0.05; NF-κB, *p* < 0.01).

#### 2.3.4. Hypoxia-Inducible Factor 1α (HIF-1α) Expression

**Figure 7:**
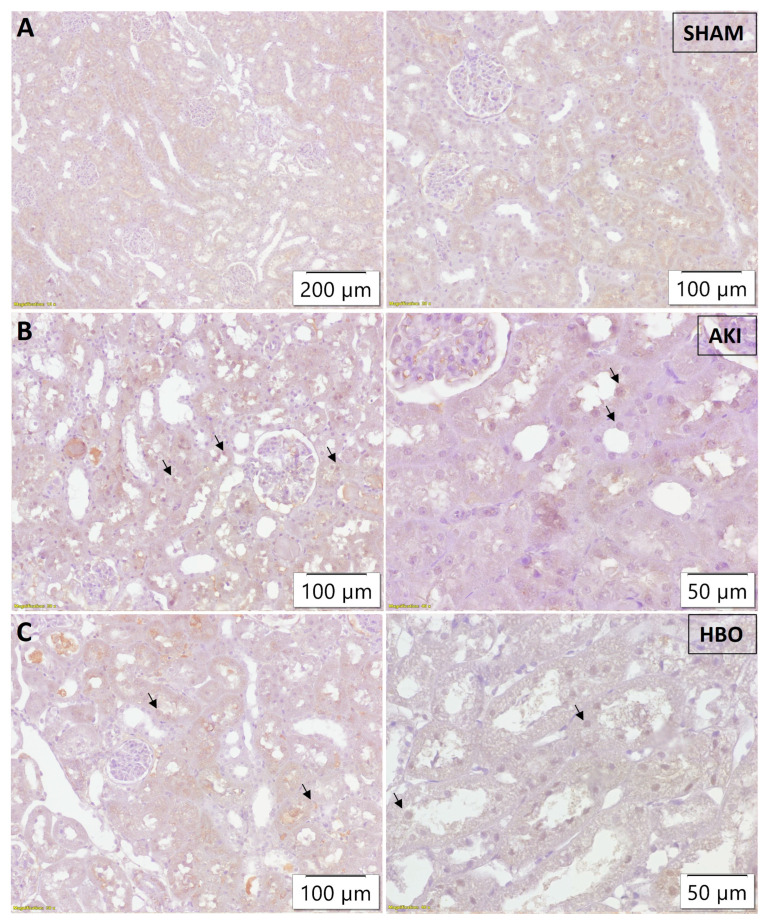
Immunohistochemical HIF-1α expression in representative kidney samples collected in different experimental groups: SHAM (**A**), AKI (**B**), HBO (**C**), and tubular nuclear expression (arrows).

In the SHAM group, the expression of HIF-1α was of moderate intensity, mostly in the cytoplasm of the tubular epithelial cells, with minimal expression in other kidney structures (Figure 7A). In response to hypoxic conditions after AKI induction, the expression pattern was different considering that previously noticed in the SHAM group, with HIF-1α translocation, and we observed both nuclear and cytoplasmatic expressions in tubular cells (Figure 7B). By observing the results of the immunohistochemical HIF-1 expression in the AKI + HBO group (Figure 7C) and comparing them with those of the AKI group, it was not clear if HBO preconditioning affected HIF-1 tissue expression, and in order to evaluate those results, we also performed a Western blot analysis to quantify the HIF-1α expression.

### 2.4. Western Blot Analysis of Hypoxia-Inducible Factor 1α (HIF-1α) Expression

**Figure 8:**
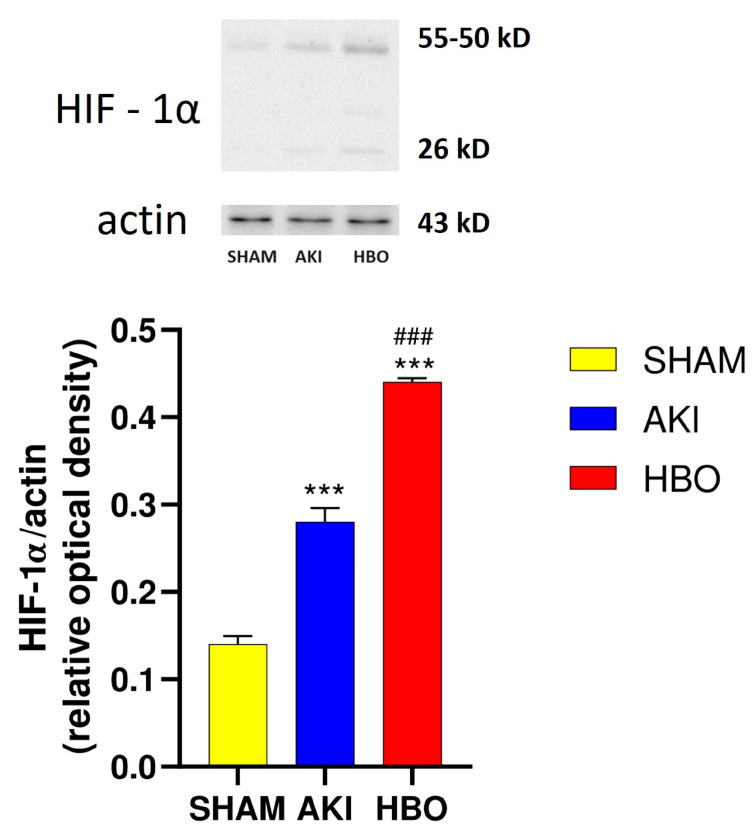
HIF-1α expression in different experimental groups: SHAM, AKI, and HBO. One-way ANOVA with Tukey’s post hoc test: *** *p* < 0.001 vs. SHAM group; ### *p* < 0.001 vs. AKI group.

The Western blot analysis showed significantly increased HIF-1α expression after AKI induction in comparison to the SHAM-operated rats (*p* < 0.001). Nevertheless, the expression was even more pronounced in the group with hyperbaric oxygen preconditioning compared to the AKI group (*p* < 0.001; Figure 8).

## 3. Discussion

In animals with induced postischemic AKI, we registered a drastic drop in eGFR, which was also a confirmation that the experimental model was adequately performed. In the group of animals exposed to HBO preconditioning, we noted a significant improvement in eGFR, indicating a general protective effect of this pretreatment in postischemic AKI. Previously, we showed that HBO used before AKI induction improved the renal hemodynamic by increasing renal blood flow and decreasing renal vascular resistance [29], and this result is in the line with the improvement in eGFR that we observed in the present study. Reduced renal blood flow accompanied by tissue hypoxia has been proposed as an important factor in the pathogenesis of postischemic AKI by initiating the activation of multiple mechanisms, including increased oxidative stress, inflammation, apoptosis, DNA damage, and necrosis [14,20]. In this research, we focused on tissue oxidative stress and systemic antioxidant capacity during AKI by evaluating the effects of HBO preconditioning on 3-NT immunohistochemical expression and plasma FRAP and ABTS levels.

In the SHAM group, we detected 3-NT expression only focally in peritubular capillaries and glomeruli. It is not surprising, considering that our experimental animals were spontaneously hypertensive rats, and hypertension is accompanied with oxidative stress [32]. But in AKI, strong and abundant tubular immunohistochemical expression of 3-NT was presented, along with expression in glomeruli and peritubular capillaries, which can be related to the increase in oxidative stress during acute kidney injury. In the kidney, NADPH oxidase and the mitochondrial respiratory chain are considered to be the main sources of ROS production [33]. As the level of oxidative stress increases, ROS and RNS promote oxidative damage and cellular death [6,13], playing a very important role in pathogenesis of postischemic AKI. These molecules directly and indirectly affect all aspects of the renal function, including vascular reactivity, renal hemodynamics, glomerular filtration, tubular reabsorption, and secretion [13]. Also, oxidative stress exerts harmful effects on biomolecules, such as proteins, DNA, RNA, enzymes, and lipids. Actually, the changes that reactive species (RS) make upon these molecules could be added up and used as biomarkers of oxidative stress [34]. The nitration of tyrosine residues to 3-nitrotyrosine in proteins represents an oxidative posttranslational modification. Indeed, high levels of ROS in the presence of NO or NO-derived metabolites lead to the formation of nitrating species such as peroxynitrite [35]. As nitrotyrosine represents a specific peroxynitrite-mediated protein modification, the detection of nitrotyrosine in proteins is considered to be a biomarker of cell, tissue, and systemic “nitroxidative stress” [35,36]. Quin et al. documented that increased plasma 3-NT level was associated with the mortality of AKI patients independent of the severity of illness [37].

Our results showed that HBO preconditioning reduced both the intensity and the extent of 3-NT immunohistochemical expression in all kidney structures compared to AKI. This finding was accompanied by increased plasma antioxidant capacity, as estimated by FRAP. These data may be interpreted as a helpful effect of HBO preconditioning, as it, at the same time, decreased oxidative stress in renal tissue and increased plasma antioxidant defense. These results are in accordance with our previous studies, obtained on the same experimental model, in which we showed that HBO used as pretreatment decreased other parameters of oxidative stress, plasma TBARS level, marker of lipid peroxidation in plasma [29], and renal tissue expression of 4-hydroxynonenal (4-HNE) compared to AKI group [31]. At the same time, plasma glutathione reductase activity, an enzyme that is involved in antioxidant response, was increased [29]. As it was discussed above, increased oxidative stress, accompanied by high levels of ROS and RNS, induces many damaging effects, but, on the other hand, ROS and RNS are able to trigger signaling processes at low levels of oxidative stress [13]. It was shown that hyperbaric oxygen is also an important stimulus to upregulate antioxidant enzymes in response to a greater production of ROS [38,39]. We can assume that intermittent hyperoxia, induced by HBO preconditioning, in our study, provokes a “suptile increase” in ROS that further activated signaling pathways, including upregulating antioxidant enzymes. This can contribute to decreased oxidative stress in postischemic AKI upon HBO preconditioning, which we observed in present study. It may sound paradoxical that HBO decreases oxidative stress by inducing ROS formation, but ROS may have both a physiological and pathophysiological role, depending on the level of ROS that is produced. In addition, increased ROS is associated with enhanced renal vascular resistance [40], so we can presume that decreased oxidative stress and increased antioxidant capacity after HBO preconditioning may decrease renal vascular resistance that would further improve the renal hemodynamic [29] and glomerular filtration rate, as we showed in our research by evaluating eGFR.

DNA damage, including double-strand breaks, may be the major result of oxidative stress [41]. The immunohistochemical pattern of γH2AX(S139) histone expression in the SHAM group can be explained by the literature data indicating that the cells in aging organisms and senescing cells in culture display an increased γH2AX(139) signal even if there is no intentional damage [42,43]. This immunohistochemical pattern was maintained in the AKI group, but a much higher extent and intensity of γH2AX(S139) was observed. However, HBO preconditioning led to an evident reduction in γH2AX(S139) tissue expression, highlighting, again, its beneficial effect on AKI.

We observed a low expression of HIF-1α in SHAM operated animals, but in response to hypoxic conditions after AKI induction, we detected increased nuclear and cytoplasmatic expression in tubular cells, in contrast to the SHAM, where HIF-1α was observed mostly in the cytoplasm. These results are in accordance with previously published data. HIF-1α is a key player in the transcriptional response to hypoxia. It was shown that the HIF-1α gene is constitutively active with weak expression under normoxic conditions [22], but in response to hypoxia, it is significantly increased [44]. Furthermore, HIF-1α is ubiquitously expressed [45,46]. In the kidney, HIF-1α is found in most renal epithelial cells, also detected in endothelial and interstitial cells of the papilla and inner medulla, but not in the outer medulla and cortex [47].

It should be noted that, when an ischemic tissue that overexpresses HIF is exposed to HBO therapy, tissue hypoxia is corrected and reversed, and, consequently, the overexpressed HIF is reduced towards the baseline [48,49,50,51,52]. In this study, we showed that hyperbaric oxygenation preconditioning significantly increased HIF-1α. This result can be explained by the already known hyperoxic–hypoxic paradox, actually with the fact that, in the cellular environment, variations in free oxygen levels rather than the absolute value of oxygen are interpreted as a deficiency of oxygen [22]. Duan et al. and Gu and colleagues showed, in their studies, separately, that when HBO is used as preconditioning, by HIF induction, the tissue will tolerate the ischemic insult better, harmful effects induced by ischemia will be alleviated, and the overall post-insult HIF expression will be higher than expected for the insult itself [53,54]. Hypoxia and inflammation are closely related [55]. The literature data indicate crosstalk between NF-κB and HIF-1α. Various studies have shown that NF-κB upregulates HIF-1α [56,57,58], but whether HIF-1α can directly regulate NF-κB in AKI is a matter of debate [59]. Activation of NF-κB may be induced as a protective compensatory tissue response, but increased and prolonged NF-κB activation is followed by abundant proinflammatory cytokines and can significantly contribute to tissue damage. In this study, we presented that increased HIF-1α renal expression detected in AKI + HBO group was followed by reduced NF-κB expression compared to animals with induced postischemic AKI but without HBO preconditioning. These results are in accordance with some preclinical and cell cultured studies that evaluated the effects of hyperbaric oxygen on NF-κB pathways. Bandarra et al. demonstrated that HIF-1α could limit NF-κB transcriptional activity in vivo and in vitro under inflammatory conditions [55]. In 2018, Liu and colleagues found that hyperbaric oxygen inhibits neuroinflammation through the inhibition of the lipopolysaccharide-induced NF-κB/mitogen-activated protein kinases–CCL2/CXCL1 signaling pathways [60]. Yu et al. observed that hyperbaric oxygen reduced the inflammatory response in acute pancreatitis by inhibiting NF-κB activation [61]. Vinkel and coworkers, in their review, summarized that, during normoxic homeostatic conditions, hyperbaric oxygenation increases both HIF-1α and NF-κB levels. On the contrary, during hypoxia, HBO exposure decreases HIF-1α and NF-κB signaling. They also pointed out that, in cases of inflammation followed by relative hypoxia, the effects on HIF-1α depend on the number of HBO sessions, whereas the effect on NF-κB expression, primary decreases, which indicates that multiple mechanisms impact NF-κB signaling during inflammation [62]. In our study, we used HBO as a preconditioning over a period of two days; more precisely, the experimental animals were exposed to HBO in normoxic homeostatic conditions, twice a day, 12 h apart; and 24 h after their last HBO session, they were exposed to severe hypoxic conditions by inducing postischemic AKI. So, decreased NF-κB renal expression, as we observed in the present study, is a result of net effects induced by multiple normoxic/hypoxic interactions on the NF-κB signaling pathway. Despite the fact that many researchers have investigated the effect of HBO on NF-κB activation, the precise mechanism by which oxygen sensing regulates the NF-κB pathway remains unclear [62]. On the other hand, there is a fine interplay between NF-κB and nuclear factor erythroid 2-related factor 2 (Nrf2). Nrf2 is a transcriptional regulator of cellular defense against oxidative stress that has been shown to improve renal damage by eliminating ROS [63], and it is also considered to be a therapeutic target for AKI [64]. It is documented in several papers that HBO’s protective effect is mediated by the activation of Nrf2 signaling pathways [65,66,67]. In addition, it was confirmed that the return to normoxia after 100% O_2_ hyperoxia exposure induces a shift towards an oxidative stress response followed by an Nrf2 and NF-κB increase in the first 24 h post-exposure [68,69]. In our study, the same exposure dose of oxygen led to an NF-κB decrease, but in the hyperbaric condition. Balestra et al. [69] illustrated that variations in ROS production were not directly dependent on the oxygen dose up to 48 h following 60 min of exposure and emphasized that “the dose seems to not be the only clue”. Later, in another publication, it was also pointed out that pulsed high oxygen treatment provokes specific cellular response according to its partial pressure and time of administration [70]. Nrf2 controls the expression of cytoprotective genes, including heme oxygenase-1 (HO-1), [71]. Previously, we showed that HBO preconditioning upregulates HO-1 expression in experimental model of postischemic AKI [30], but we need further study to evaluate is this HO-1 upregulation accompanied with increased Nrf2 expression itself.

Upregulation of HIF-1α during hyperbaric oxygenation preconditioning can be considered as its protective and useful effect on postischemic AKI. HIF-1 and HIF-2 stimulate multiple cellular and tissue responses that are involved in cellular adaptation to hypoxia, including anaerobic glucose metabolism, iron metabolism, erythropoiesis, angiogenesis, NO and adenosine metabolism [72,73]. Besides this, many studies on experimental models suggest HIFs take part in kidney repair. Appropriate kidney repair depends on a precise balance between renal tubular cell death and proliferation [74]. HIF-1α affects cell death by regulating Bcl-2 family genes, interacting with p53, and/or targeting mitochondria enzymes [75]. A large amount of work in different kinds of AKI models has demonstrated that HIF activation decreases renal apoptosis [76,77,78]. As we have already showed that HBO preconditioning in experimental model of postischemic AKI induction increases renal HO-1 and antiapoptotic Bcl-2 protein expression [30], we may say that there are multiply mechanisms that are responsible for helpful effects of HBO on pathogenesis of AKI, besides physical increase in free oxygen in blood and tissues. We also want to underline the importance that in this research we showed protective effects of HBO preconditioning in spontaneously hypertensive rats, considering high incidence of hypertension worldwide, as well as the fact that hypertension is risk factor for many other acute and chronic disease, including acute kidney injury.

Pharmacological activation of HIF-1α, by inhibition of prolyl hydroxylase domain-containing protein (PHD), which mediates degradation of HIF, applied as a pretreatment, significantly alleviates ischemic kidney injury by upregulating HIF target genes, followed by reducing apoptosis, macrophage infiltration, and vascular cell adhesion molecule 1 (VCAM1) expression [76,77,79,80,81]. Thus, upregulation of HIF abundance by PHD inhibitors is an attractive potential therapeutic target [82]. However, the effect of pharmacological activation of HIF-1α and HIF-2α as a postischemic treatment is controversial [23]. In remnant kidney model, pharmacological activating HIF ameliorated tubulointerstitial injury and decreased fibrosis [83,84].

Insufficient repair post-AKI leads to renal interstitial fibrosis [85]. Besides, HIF signaling may promote renal fibrosis via different mechanisms including epigenetic regulation, transcriptional regulation of fibrogenic genes, but also can crosstalk with other pro-fibrotic signaling pathways such as TGF-β, NF-κB, Notch, and PI3K/Akt pathways [86]. Taken together, these studies indicate that HIF takes part in renal fibrogenesis as a regulatory molecule. However, whether HIF is pro- or anti-fibrotic may depend on which, where, and when HIF is activated [23]. In addition, excessive activation of HIF may sometimes have adverse effects. It is therefore important to optimize the degree, timing, and duration of HIF activation [23].

At the end of this discussion, we would like to mention certain limitations of this study. Oxidative stress damage was evaluated through plasma FRAP and ABTS parameters and 3-NT renal expression. In order to obtain a more comprehensive analysis and assessment of the effects of hyperbaric oxygen preconditioning on the oxidative stress in postischemic acute kidney injury, it is necessary to analyze more parameters related to oxidative stress and antioxidant defense, and their potential interplay in this experimental setting.

## 4. Materials and Methods

### 4.1. Animals

For the purposes of this experiment, we used male spontaneously hypertensive rats (SHR), 24 weeks old and about 300 g an average weight, raised at the Institute for Medical Research, University of Belgrade. The animals were kept in controlled laboratory conditions (constant temperature, 22 ± 10 °C; humidity, 65 ± 1%; 12 h light/dark cycle) and fed with standard food for laboratory rats (Veterinary Institute Subotica, Subotica, Serbia), with free access to food and water.

### 4.2. Experimental Design

Before the animals were classified into experimental groups, hypertension was confirmed by indirect blood pressure measurements on a Physiograph Four device (Narco Bio Systems INC, Houston, TX, USA). After that, the animals were randomly divided into 3 experimental groups: sham-operated animals (SHAM, *n* = 6), a group of animals in which postischemic acute kidney injury was induced (AKI, *n* = 6), and a group of animals that after pretreatment with hyperbaric oxygen underwent postischemic AKI (AKI + HBO, *n* = 6).

Animals in AKI + HBO group were placed in a chamber specially adapted to the experimental conditions (Holywell Neopren, Belgrade, Serbia), where they were exposed to 100% oxygen, over a period of two days, according to the following protocol: 10 min of slow compression, exposure to values of 2.0 absolute atmospheres (ATA) during 60 min and 10 min of slow decompression, twice a day, 12 h apart. This protocol corresponds to the hyperbaric oxygenation treatment that is standardly applied in clinical conditions at the Center for Hyperbaric Medicine in Belgrade, Serbia [87], and is in the line with the recommendations of a relevant international organization, The Committee of the Undersea and Hyperbaric Medical Society [88].

Each exposure took place at the same time in order to exclude possible variations caused by changes in the biological rhythm. After pretreatment with hyperbaric oxygenation, the animals’ body temperature was measured, and no significant change was observed after the HBO pretreatment. Acute kidney injury was induced in the AKI + HBO group 12 h after the last hyperbaric oxygenation exposure.

Before surgical procedures, rats were anesthetized by injecting 35 mg/kg body weight of sodium pentobarbital (Sigma Aldrich, St. Louis, MS, USA), intraperitoneally. After the laparotomy, which was performed through an abdominal incision, AKI was induced by removing the right kidney and occluding by atraumatic clamping of the left renal artery for 45 min with an atraumatic clamp. In the group of SHAM-operated animals, the same surgical procedure was performed, but without occlusion of the left renal artery. After the surgical procedure was completed, the abdominal incision was closed with a suture, and the animals were treated with the analgesic diclofenac. After that, the animals were placed in metabolic cages for 24 h, with free access to food and water.

### 4.3. Collection of Samples

Blood samples were taken (by abdominal aortic puncture), 24 h after AKI induction, and collected in tubes containing lithium heparin (Sigma-Aldrich, St. Louis, MO, USA) for further analysis. The collected blood was centrifuged for 20 min at 4000 revolutions per minute (Heraeus Megafuge 1.0 R, Heraeus, Hanau, Germany) in order to separate the plasma. The obtained plasma sample was stored at −20 °C until the moment of analysis.

Animals were sacrificed using pentobarbital (Sigma-Aldrich, St. Louis, MO, USA). For the determination of structural changes and immunohistochemical analysis, kidney tissue was taken immediately after sacrifice and then prepared for histological examination.

### 4.4. Glomerular Filtration Rate

All biochemical parameters for the estimation of glomerular filtration rate (eGFR), as a marker of kidney function, were measured by the automatic COBAS INTEGRA 400 plus (Hoffmann-La Roche, Penzberg, Germany) analyzer, using commercial kits: creatinine concentration—via spectrophotometric method for plasma (CREJC test cassettes for plasma, Roche Diagnostics, Germany); urea concentration—via spectrophotometric method for plasma (UREL test cassettes for plasma, Roche Diagnostics, Germany);

In order to calculate eGFR, the following formulas [89] were used:

eGFR  =  880 × W^0.695^ × C^−0.660^ × U^−0.391^ (if plasma creatinine < 52 µmol/L),

eGFR  =  5862 × W^0.695^ × C^− 1.150^ × U^− 0.391^ (if plasma creatinine ≥ 52 µmol/L),

where eGFR is the estimated GFR (µL/min), W is the weight (g), C is the plasma creatinine concentration (µmol/L), and U is the plasma urea concentration (mmol/L).

### 4.5. Determination of Parameters of Antioxidant Protection

#### 4.5.1. Ferric Reducing Antioxidant Capacity of Plasma

We used ferric reducing antioxidant power (FRAP) assay to determine the antioxidant capacity of plasma [90]. In order to prepare FRAP reagent, 25 mL of 300 mM acetate buffer (pH 3.6), 2.5 mL of 10 mM TPTZ (2,4,6-tri(2-pyridyl)-s-triazine) dissolved in 40 mM HCl, and 2.5 mL of 20 mM FeCl_3_ were mixed. Then, 70 µL of plasma was added to prepared FRAP reagent and incubated for 5 min, at 25 °C. We measured absorbance at a 593 nm wavelength. Results were expressed in mmol/L (millimoles of Fe^2+^ per liter of plasma). Vitamin C and butylated hydroxytoluene (BHT) were used as positive controls.

#### 4.5.2. Trolox Equivalent Antioxidant Capacity of Plasma

Antioxidant capacity of plasma was also determined via a Trolox equivalent antioxidant capacity (TEAC) assay (also known as ABTS assay), according to the previously described method [91]. In short, 5 mL of 7 mM ABTS (2,2′-azino-bis(3-ethylbenzothiazoline-6-sulfonic acid)) solution was mixed with 0.1 mL of 125 mM K_2_S_2_O_8_ (potassium persulfate) solution and left to stand in the dark for 12–16 h. Then, 50 mM of phosphate-buffered saline (pH 7.4) was used to adjust the absorbance of the ABTS^•+^ solution to 0.70 at a 734 nm wavelength. After that, 2.0 mL of diluted ABTS^•+^ reagent was added to 20 μL of plasma and incubated at 30 °C for 6 min. The absorbance was measured at a 734 nm wavelength. Results were expressed in mmol/L (millimoles of Trolox equivalents per liter of plasma). Vitamin C and BHT were used as positive controls.

### 4.6. Immunohistochemical Analysis

Immunohistochemistry was applied on formalin-fixed paraffin-embedded kidney samples. Four-micrometer-thick paraffin sections proceeded to deparaffinization and hydration steps, and afterwards they were introduced to heat-induced antigen retrieval in citrate buffer (pH 6.0). Novolink™ Polymer Detection System components (Leica Biosystems, Wetzlar, Germany) were applied according to manufactural instructions for immunohistochemistry protocol. Peroxidase block (5 min incubation time) and protein blocks (5 min) were applied prior to incubation with primary antibody for 1 h at room temperature. The following primary antibodies (Abcam, Cambridge, UK) were used: anti-3-Nitrotyrosine (3-NT, 1:100), anti-gamma H2A.X (phospho S139) (1:1000), anti NF-κB (1:100), and anti-HIF-1 alpha (rabbit polyclonal, 1:100). Secondary antibodies were applied from Novolink™ Polymer Detection System Kit (Leica Biosystems, Wetzlar, Germany). This Kit Detection System supplied “ready to use” post-primary antibody, and according to its manufactural protocol, it was incubated for 30 min at room temperature, followed by application of Novolink™ Polymer for 30 min at room temperature. Visualization of antigen–antibody reaction by 3,3’-diaminobenzidine (DAB) was applied for 5 min (brown products). Subsequent counterstaining with hematoxylin (30 s) was conducted. Slides were evaluated using the light microscope BX53 with DP70 camera (Olympus, Hamburg, Germany). The evaluation was performed by two independent pathologists, who were blind to the experimental information.

For the immunohistochemical scores, the intensity and extent of expression in renal structures were observed, and the mentioned parameters were scored semiquantitatively: expression intensity, on a scale from 0 to 3 (0—no expression; 1—weak expression; 2—moderate expression; 3—strong expression); and extent of expression, also on a scale from 0 to 3 (0—no expression; 1—focal expression; 2—focal to diffuse expression; 3—diffuse expression). For each of the mentioned parameters, the sum of scored changes represented the immunohistochemical score, which was compared between groups.

### 4.7. Western Blot Analysis

Kidney tissue samples were taken from six rats per group and homogenized in lysis buffer [92], and with an equal amount of protein, they were separated by 10% SDS-PAGE and transferred to nitrocellulose membrane. The membranes were incubated with the primary antibodies: HIF-1 alpha (1:1000, H6536 Sigma-Aldrich) and actin (1:500, A5060 Sigma-Aldrich). This was followed by incubation with horseradish peroxidase-conjugated secondary antibodies. Protein bands were visualized using enhanced chemiluminescence reagent and quantified by Image Lab (Bio-Rad Laboratories, Hercules, CA, USA).

### 4.8. Statistical Analysis

All data were expressed as the mean ± standard deviation (SD). A statistical analysis of each parameter of interest was carried out in the form of an analysis of variance (one–way ANOVA). When a significant F value in the one–way ANOVA test (*p* < 0.05) was obtained, Tukey’s post hoc test was used. All statistical calculations were performed using GraphPad Prism for Windows (Version 7.0, GraphPad Software, La Jolla, CA, USA).

## 5. Conclusions

HBO preconditioning significantly increased the plasma antioxidant capacity estimated by FRAP and reduced the immunohistochemical expression of 3-nitrotyrosine and γH2AX(S139) histone. These data were accompanied by a significant increase in HIF-1α and decrease in NF-κB expression in kidney tissue. Thus, HBO preconditioning, at the same time, decreased oxidative stress tissue impairment and DNA damage and increased antioxidant capacity and HIF-1α transcriptional factor. Taking all of these results together, we may conclude that HBO preconditioning has beneficial effects on acute kidney injury induced in spontaneously hypertensive rats. These results are promising and open pathways for further experimental and clinical studies to confirm the protective effects of HBO preconditioning.

## Data Availability

The data presented in this study are available within the article.
